# Medical Education—The State License to Practise Medicine1Read at the ninety-second annual meeting of the Medical Society of the State of New York, at Albany, January 25, 1898.

**Published:** 1898-04

**Authors:** William Warren Potter

**Affiliations:** Buffalo, N. Y., Examiner in obstetrics and president of the State Board of Medical Examiners representing the Medical Society of the State of New York


### MEDICAL EDUCATION—THE STATE LICENSE TO PRACTISE MEDICINE.[1]

By WILLIAM WARREN POTTER, M. D., Buffalo, N. Y.,
Examiner in obstetrics and president of the State Board of Medical Examiners representing the Medical Society of the State of New York.

THE first proposition of which there is record to separate the teaching and licensing powers in this state came from the medical faculty of the university of Buffalo, as the following minute taken from the records of the college will attest:

University of Buffalo, Medical Department,
February 2, 1864.

On motion of Professor Charles A. Lee, seconded by Professor James P. White, it was

*Resolved*, That the medical society of the state of New York be requested to appoint a committee to consider the expediency of, and to report a plan for, the appointment of a state board of examiners for the degree of doctor of medicine, and to report at the next meeting of the society.

*Resolved*, That the same committee be instructed to bring the subject before the next meeting of the American medical association, and that the delegates of this society be instructed to urge the general adoption of the same plan in other states of the union. Carried unanimously.

THOS. F. ROCHESTER,

*Chairman.*

Sandford Eastman,
*Dean of the Faculty.*

The Buffalo Medical Journal, then edited by Dr. Julius F. Miner, a member of the college faculty, in its issue of February, 1864, commented on the suggestion as follows:

When the graduation of students in medicine is wholly separated from the duty of teaching and an impartial board of examiners shall decide who shall and who shall not receive the degree of doctor in medicine, very much will be accomplished for the elevation and advancement of the profession. It is striking at the very root of a great evil

---

1. Read at the ninety-second annual meeting of the Medical Society of the State of New York, at Albany, January 25, 1898.

and will meet with opposition; indeed, we have no doubt it will be overwhelmed in the almost unanimous opposition which it will meet from the various institutions now empowered to grant diplomas. . . . . . If this reform now suggested and urged upon the profession by the medical college in Buffalo is favored by the other colleges in the state, we shall soon be redeemed from the power and influence of a system which has disgraced the profession, lowered its standard of attainment, reflected obloquy and contempt upon its degree and come well-nigh reducing medicine as learned and practised to the level of a trade.

The JOURNAL's predictions were well founded, for the scheme failed, as it never went beyond the portals of this society.

---

In 1883, the project was again suggested, this time also in Buffalo. The medical society of the county of Erie, at the instance of Dr. Henry Reed Hopkins, formulated a bill and sent it to this society for approval and formal presentation to the legislature. Here it was debated a year or two and after amendment was finally offered to the legislature. I need not recount the vicissitudes it therein encountered, for the circumstances are yet fresh in the minds of many here present.

After repeated defeats the bill in amended form finally passed both houses, receiving the signature of the governor, June 5, 1890. The law provided for three separate boards of medical examiners, one to represent the medical society of the state of New York, one to represent the homeopathic medical society of the state of New York, and one to represent the eclectic medical society of the state of New York. Each of these societies was directed to send the names of fourteen or more of its members to the regents of the university of the state of New York, from which the regents were directed to appoint a board of seven members to represent each of these several societies.

The law further provided that these boards should organise for duty on or before September 1, 1891, the date when the act should go into effect.

---

On March 11, 1891, the regents appointed to represent this society as an examining board, in accordance with the law, the following-named examiners: for three years, Dr. William Warren Potter, of Buffalo, Dr. William S. Ely, of Rochester, and Dr. Maurice J. Lewi, of Albany (now in New York); for two years,

Dr. William C. Wey, of Elmira, and Dr. George Ryerson Fowler, of Brooklyn; for one year, Dr. Eugene Beach, of Gloversville, and Dr. J. P. Creveling, of Auburn. The names are here given in the order of their announcement by the regents.

The examiners met in the capitol at Albany, September 1, 1891, and organised as a board by electing William C. Wey president and M. J. Lewi secretary. Dr. Lewi and Dr. Fowler were appointed members of the question committee. The law provided for examination in seven topics and these were distributed among the examiners as follows: anatomy, Dr. Ely; physiology and hygiene, Dr. Wey; chemistry, Dr. Lewi; surgery, Dr. Fowler; obstetrics, Dr. Potter; pathology and diagnosis, Dr. Creveling; therapeutics, practice of medicine and materia medica, Dr. Beach.

---

During the winter of 1891, the medical students throughout the state succeeded in persuading the legislature to pass a bill exempting all who began the study of medicine before June 5, 1890, from the operations of its provisions. Under this amendment about 900 students were relieved of the state examination. Another attempt was made the following year on the part of the students to procure a further exemption from the provisions of the law, but this was not successful. However, the previous wholesale exemption bill deprived the board of the major part of its anticipated work for more than two years; for it was not until 1894 that our work really began to be effective.

---

Many attempts have been made to so modify the law as to depreciate its effectiveness as a reform measure in medical education; and while in some instances they have almost succeeded, up to the present the original law is in practical force. It has been once or twice amended at the instance of the regents in order to make its provisions more harmonious and effective.

Last year we had two narrow escapes. It is a well-known fact that the members of the New York state medical association, individually or collectively, opposed from the outset the present practice act; sometimes, indeed, theirs has been the principal antagonism, either to amendments calculated to strengthen it or in proposing amendments that would weaken or emasculate it; or in embarassing its operations in minor ways.

The attempt was made during the legislative session of 1897 to so amend the law as to compel the regents to select one-half the examiners from that association. If a numerous array of its members at the hearing before the committee; if a forceful presentation of a false premise; or if lurid rhetoric, redolent metaphor and abusive syntax ever deserved to succeed it would seem as if their opportnity had come on the occasion referred to. The little coterie that defended the interests of this society, however, were fortunate in appearing before a singularly intelligent committee, where it proved not difficult to again demonstrate that "Thrice is he armed that hath his quarrel just."

It was, indeed, a fortunate escape, and the impression has been left behind that the attempt will be renewed, probably this present year. Will this society stand idly by and witness the consummation of such an outrage upon its vested rights—to say nothing of the violation of the proprieties of the occasion—as such an amendment would entail? If it would prevent it, a most thorough system of defense must be organised and that speedily. The legislative committee must not be left to fight the battle alone, but a strong phalanx of able and active men—members of this society—must attend at the capitol, if summoned in an emergency.

We escaped another dilemma through a still narrower passage. In spite of the utmost watchfulness a bill passed both houses, which in effect compelled the recognition of licenses of other states, no matter what their standards. It slipped through in the last days of the session, when bills may be passed without the ordinary scrutiny of a committee. Fortunately, however, Governor Black, recognising the dangerous character of the measure, included it in his excellent veto list.

---

This brings me to the consideration, for a moment, of the recognition of other state licenses. Undoubtedly, it would be most desirable if all the state licenses were interchangeable or interindorsable, for then a licensee of any state could roam all over the country with a free lance. It so happens, however, though twenty-eight or more states grant licenses, that no two have in all respects an equal standard. Some of them merely visé a legal diploma; others examine only candidates that have no diplomas; still others require no preliminary education; while a few grant licenses only to those who give proof of preliminary education, three or four years of medical college teaching and a diploma—to which latter class belongs New York.

To exchange courtesies by granting interstate indorsement of licenses to those from states where standards are lower than ours would be a manifest injustice, a discrimination against our own citizens.

---

As a remedy, it has been proposed in certain quarters to abolish state examining boards and establish in their stead a national board; but a moment's thought will convince any reflecting mind of the impracticability, not to say impossibility, of carrying out such a scheme. The states will not surrender the right to make their own police regulations, nor will the general government deprive the states of their vested constitutional privileges.

The real remedy consists in establishing uniform standards of requirements in all the states. This should embrace the triad—preliminary education, medical collegiate training, and state examination for license. It would not, or at least ought not to be difficult for the states to determine that a high school diploma, or its equivalent, shall be the minimum of preliminary requirement ; and that without this the schools shall not receive students. Colleges should then be required to establish four years' courses and, finally, a state examination of uniform standard in all the states, no candidate to be admitted to it unless he presents a diploma from a school recognised as maintaining the proper preliminaries for graduation.

A national clearing house made up of representatives from the several states could pass upon or visé the diplomas of such as desire to change states—such visé to entitle a diploma to registration in the state in which its holder desires to take up residence. While there are many opinions on this vexed question, most of which are made up from special cases of so-called hardship, there really remains but one equitable solution of the problem and that is for the several states to establish uniform standards of preliminaries, collegiate courses and state examinations.

---

The character of the examination imposed by the state has been subject of criticism. One critic would ask simpler questions ; another would make them more severe; still another says the questions are too bookish, or are not practical in character ; yet another would institute a clinical or bedside examination in the hospital wards ; while a fifth would have us divide the examination, making an undergraduate test at the end of two years in anatomy, physiology and chemistry. It is easy to find fault, but difficult to attain the ideal. The fourth proposition is well nigh absurd because, first, it cannot be carried out, and, second, it is unnecessary. We are examiners, not teachers, and our province is simply to determine the nature and value of the instruction candidates have already received and whether it adequately fits them to practise. These latter reasons will apply equally well to the proposition to divide the examinations.

It must not be forgotten that while our law provides for three examining boards, it also says their standards must be alike, except as to therapeutics. In order to attain a common level, it has been found necessary to appoint a question committee, consisting of two members from each board. This committee practically becomes a clearing house, where all questions are scrutinised and finally made up into groups in each topic for the several examinations. It will thus be seen that an examiner is liable to find questions credited to his topic that he did not furnish and, perhaps, to which he himself objects. This makes him apparently responsible for a group of questions at a given examination that, as a whole, he did not propound—a condition of things that appears somewhat incongruous. Nevertheless, it serves to maintain a standard of uniformity in all the boards; and I venture to assert that, in the main, the questions of the New York boards take an average rank or standing with any propounded at the schools or by other examining boards.

On the whole, our examination is regarded by medical teachers of high standing and by other state examining boards as a post-graduate test of a creditable nature. Candidates, too, have almost invariably commended it for fairness, thoroughness and excellence. The few that have complained have been imperfectly trained in the schools and have not been properly equipped to practise medicine. Even many who have been rejected praise the quality and impartiality of the test. College teachers in general have become our strongest allies and would not go back to the old way if they could.

---

Permit me to quote a letter from a distinguished teacher, which is a type of many similar statements, verbally or in writing, made to me on the subject. The letter reads in part as follows:

The influence of the state examination upon our institution has been most beneficial. In proportion as the requirements have been increased in kind and numbers we have had a larger attendance as well as a better class of students. The advantages of teaching educated men, as compared with the more ignorant students of previous years, are apparent to every teacher, in that we are no longer required to condense and simplify our lectures, but we can go deeply into the subjects proper and do better and more advanced work. This is an advantage both to the student and the teacher and is telling in the better class of graduates which we are able to turn out.

JOHN PARMENTER,
*Secretary Faculty Med. Dept., University of Buffalo.*

While the present system is commendable in guarding the entrance doors to medicine, our laws or their administration are imperfect or inadequate to control or prevent itinerant quacks, christian-science mountebanks, galvano-magnetic fakirs and other illegal practitioners from preying upon an unwary public.

In the county of New York it is true that, under the vigilance of a splendidly organised medical society, many convictions have been secured and a large sum in fines has been collected, yet in the county of Erie not one prosecution has even been undertaken. The prosecuting attorney of the county seems loath to proceed on the evidence furnished by the board of censors. Surely a law that is adequate to bring dismay to its violators in New York ought to be equally potent in Erie !

---

In Kentucky, where the law relating to medical education is much inferior to ours, it has been found stout enough to drive 1,150 irregulars from the state, so that now it is not infested by a single illegal practitioner of medicine, nor can such regain a footing within its territory. The profession of the blue grass state is to be congratulated upon this splendid record and upon the example it has set for other commonwealths.

---

It has always seemed to me that it would be a good plan if our college teachers would carefully inform their pupils as to the working of the practice law. Let students be told that the state examination is one of the proper safeguards erected by the states for the protection of the people against fraud and incompetency ; and that the state license is a prize to be won in the race for professional standing and not a *bête noir*—not something to be dreaded and to be resisted. We try to make our examinations fair and practicable, free from catch questions and to represent a test that demands only such knowledge as any and every candidate ought to possess before offering his services in behalf of the sick or injured.

---

It is interesting to observe what has been accomplished by the act creating state boards of medical examiners. In 1890, two years of collegiate training were sufficient to entitle a candidate to a degree. Nor were there any restrictions or regulations as to preliminary requirements; that entire question was left to the colleges themselves and they generally looked with indifference upon an applicant's literary attainments or want of such. Finally, there were no examinations for state license, hence each possessor of a legal medical diploma, no matter how obtained, might offer his professional services to an unwary public.

On the other hand, the medical graduate of 1898 must at least be a good English scholar, must have had three or four years of college work and must successfully pass the state board with an average standing of 75 or more points.

Since September 1, 1891, the state boards have examined 3,103 candidates and have rejected 704 or 22.68 per cent. When it is remembered that each one examined had already received the indorsement of the schools, it can easily be determined what is the significance of the work of the boards. But more than this: the colleges themselves have become more conservative. I know an instance in which a college faculty turned back twenty candidates in its class of '97. How many of these, think you, would have been refused diplomas in 1890?

A further factor to be taken into account is the rejection by the regents' office of a large number of informal applications for license from persons unable to meet the requirements. A conservative estimate of such applications and rejections, as shown by the records of the regents' office, would swell to at least 30, the per cent. of the whole number of applicants that have failed to obtain the license of the state of New York to practise medicine.[1]

---

When the legislature can be persuaded to recognise no propositions to amend the present law that do not originate in this society or the regents' office, and when it can be prevailed upon to so

1. Regents' Examination Bulletin, November, 1897.

strengthen the statute as to suppress every illegal practitioner within the borders of the state, then can we truly feel that a great work has been accomplished, leaving the medical profession of the empire state above suspicion and beyond reproach.

284 Franklin Street.

